# Community assets and multimorbidity: A qualitative scoping study

**DOI:** 10.1371/journal.pone.0246856

**Published:** 2021-02-24

**Authors:** Maria Kordowicz, Dieu Hack-Polay

**Affiliations:** 1 Department of Management, Lincoln International Business School, University of Lincoln, Lincoln, United Kingdom; 2 School of Population Health & Environmental Sciences, Faculty of Life Sciences & Medicine, King’s College London, London, United Kingdom; Anglia Ruskin University, UNITED KINGDOM

## Abstract

Little is known of how community assets can play a role in multimorbidity care provision. Using a rapid ethnographic approach, the study explored perceptions of the role of community assets in how multimorbidity is managed within Southwark and Lambeth in Southeast London, England. The scoping work comprised of four micro-studies covering (1) Rapid review of the literature (2) Documentary analysis of publicly available local policy documents (3) Thematic analysis of community stories and (4) Semi-structured stakeholder interviews. The data were analysed using framework thematic analysis. Themes are presented for each of the microstudies. The literature review analysis highlights the role of attitudes and understandings in the management of multiple long-term conditions and the need to move beyond silos in their management. Documentary analysis identifies a resource poor climate, whilst recognising the role of community assets and solution-focussed interventions in the management of multimorbidity. Community patient stories underline the lack of joined up care, and psychosocial issues such as the loss of control and reducing isolation. The stakeholder interview analysis reveals again a sense of disjointed care, the need for holism in the understanding and treatment of multimorbidity, whilst recognising the important role of community-based approaches, beyond the biomedical model. Recommendations stemming from the study’s findings are proposed. Upholding access to and resourcing community assets have key practical importance.

## Introduction

This rapid ethnographic study is a research scoping exercise to examine the understandings of community assets and multiple long-term conditions (MLTCs) in Lambeth and Southwark, two densely populated London Boroughs. The purpose of the study is to provide insight into community assets, multimorbidity and the dynamics of the relationship between them, harnessing learning and implications for health improvement.

Multimorbidity is defined as the presence of several chronic medical conditions [1]. These may be related to physical and/or mental health. A long-term or chronic health condition is one which lasts or is expected to last longer than three months. For the purpose of this study, MLTCs are defined as living with two of more diagnosed chronic conditions. According to the National Institute for Clinical Excellence ‘Multimorbidity matters because it is associated with reduced quality of life, higher mortality (…) health services use (including unplanned or emergency care).’ [2]. The World Health Organization (WHO) has identified long-term conditions as the leading causes of death globally and a top ten threat to global health [3].

The diverse Southeast London Boroughs of Southwark and Lambeth were apt settings for this study. Southwark is the ninth most densely populated local authority in England and Wales. Reflecting the Borough’s diversity, in the 2011 Census 54.2% of the population defined themselves as white, 26.9% as black or black British, 6.2% as mixed race and 9.4% of the population self-defined as Asian. In 2011, 18,978 residents reported that they had a long-term health problem or disability. The number of working age people with serious physical disabilities was projected to increase by 23% between 2012 and 2020 [4]. Lambeth is the fifth most densely populated local authority in England and Wales, within around 150 different languages spoken. It is estimated that 37,000 people in Lambeth have their day-to-day activities limited by a long-term illness or disability.

Community level approaches here are viewed as assets in terms of care that voluntary, not-for-profit, and community-interest organisations offer. An understanding of existing community assets can help stakeholders to build upon pre-existing community resources in the face of limited funding [5]. Several years into an austere socio-political backdrop, with significant reduction in public sector health and social care funding, community assets may provide the necessary resource to deal with the health burden of multimorbidity [6, 7]/However, little is known about the attitudes towards community assets in local populations and the extent to which they are perceived to be helpful for those living with multiple long-term conditions. Further, the extent to which it is felt that community assets have the capacity and resource to provide support for multimorbidity is unknown.

The chronic nature of MLTCs, with diagnoses typically ‘layering’ over time, may lend itself well to being understood in the context of a journey. It is known that prior to multimorbidity, patients tend to hold a diagnosis of just one condition initially [8]. Limited knowledge exists about how patients and community stakeholders and the services they represent perceive the experience of transition from a single disease to multimorbidity over the life course. Attempts at reconstructing the journey towards multimorbidity would assume that life events play into patients’ experiences of MLTCs, and there is a paucity of research exploring the meanings surrounding those life events, alongside tailored interventions at different steps on the MLTC journey. Therefore, this scoping study reviews several sources of evidence, in order to generate ideas to inform future community-based multimorbidity research and programmes.

The objectives of our study were to facilitate greater understanding of multimorbidity and community resources for multimorbidity in Lambeth and Southwark, as well as of future implications for service organisation. We achieved these objectives by investigating stakeholder and policy-maker attitudes towards community assets and their role in multimorbidity. The focus of the study was on providing a holistic capture of perceptions of community assets, rather than on individual patient experience.

## Methods

### Study design & methodology

Qualitative methodology was chosen for this study due to its primary concern with exploring attitudes and perceptions surrounding multimorbidity and community resources in Lambeth and Southwark. The underpinning epistemology centred on social construction–the notion that people’s perceptions of reality are subjective and created within and shaped by the social context [9].

The method employed was rapid ethnography, whereby several qualitative approaches are triangulated, typically in situations in which research needs to be completed within tight timeframes and is often targeted at a specific programme or pre-identified issue. Rapid ethnographies have been recognised for their popularity and utility in health services research, where fast changing priorities require operationalizable findings within a characteristically brief timeframe [10]. Given the short timeframe of this study (six months), it was felt that rapid ethnography was the most appropriate approach to build greater understanding of MLTCs and community assets by bringing together multi-source data for the scoping exercise. Indeed, rapid ethnography is characterised by “forms of intensity that lead to deep and valid ways of knowing” [11]. Rapid ethnography aims for a contextual exploration of a social and cultural setting and its issues before a deeper investigation is undertaken using traditional ethnography, which requires a longer period of time with the research participants in order to gain more detailed understanding of that socio-cultural environment.

In line with the rapid ethnography method, the research questions were explored through four micro-studies, offering a preliminary insight into multiple data sources, the findings of which are brought together in the analytical discussion. The micro-studies and the methods and process employed are explained below.

### Process

#### Literature review

A rapid literature review method was applied by sourcing qualitative literature. Rapid reviews are often used by policy-makers and recognised as providing opportunities for building an evidence-base within the time-constraints of health service delivery [12]. The aim of the rapid literature review was to locate literature and identify themes related to journeys to multimorbidity and community engagement. In this study, rapid literature review covered the MLTCs literature published since the introduction of the Health and Social Care Act 2012, which marked the start of unprecedented reforms within the health and social care sector, until the day of the search (February 2018).

In the first instance, an electronic academic literature database search focusing specifically on Southwark and Lambeth was conducted utilising various groupings of the following search terms:

‘long-term condition(s)’; ‘LTC(s)’; ‘chronic disease(s)’; ‘Lambeth’; ‘Southwark’; ‘multimorbidity’; ‘community’ (resource(s); asset(s)); ‘charity’; ‘voluntary’

Peer-reviewed journal publications and editorials were included in the review.

There was a paucity of literature concerning long-term conditions in Lambeth and Southwark; thus, literature from beyond these geographical areas was added to the review by widening the search to studies published in English language. Hence, the authors considered sources in what is often referred to as ‘grey literature’, i.e. documents (reports, policy documents, news articles, etc.) outside the mainstream academic literature. Though these may not always have the weight of tested and validated academic papers, we ensured that the ‘grey literature’ consulted was from credible sources which have been vetted [13] for example government reports, NHS publications, to supplement the limited scholarly literature available. The papers identified were brought together into a narrative with the aim of conveying the key themes from the literature pertaining to the aims of the study.

#### Documentary analysis

Publicly available local health and social care reports, guidance, presentation slides and webpage contents related to long-term conditions in Lambeth and Southwark since 2012, which cover the period since the introduction of the Health and Social Care Act 2012, were collated to create a documentary thematic framework analysis [14]. These were located through online searches using combinations of the search terms: *‘long-term condition(s)’; ‘LTC(s)’; ‘chronic disease(s)’; ‘Lambeth’; ‘Southwark’; ‘multimorbidity’; ‘community’ (resource(s);asset(s)); ‘charity’; ‘voluntary’; ‘policy’; ‘report’; ‘strategy’*

The documents collated from both Boroughs are presented in Table 1:

**Table 1:** Local policy documentary data sources.

*Age UK Lewisham and Southwark Annual Reports 2013–2016*
*Annual Public Health Reports 2012 onwards*
*Commissioning for Value Long-term Conditions Pack–Southwark CCG 2016*
*Health and Wellbeing Strategy Southwark 2015–2020*
*Healthwatch Lambeth Annual Review 2016–2017*
*Healthwatch Southwark Annual Report 2015–2016*
*Improving Health*, *Improving Quality in Lambeth*: *Lambeth Clinical Commissioning Collaborative Commissioning Strategy Plan Refresh 2012/13-2014/15*
*Integrated Care for Long-term Conditions–Southwark Presentation Slide Pack (not dated)*
*Joint Strategic Needs Assessment Reports and Documentation*
*Lambeth and Southwark Singing for Better Breathing Final Report GSTT Charity 2017*
*Lambeth Council Commissioning for personalisation*: *direction of travel (not dated)*
*NHS Lambeth Clinical Commissioning Group “Healthier Together” Five Year Strategy*: *2014/15-2018/19*
*NHS Southwark Clinical Commissioning Group (CCG) & Southwark Council Joint Mental Health and Wellbeing Strategy 2017–2020*
*Public Health Report Health in our Community–Southwark 2013–2014*
*Quarterly Reports of the Director of Public Health 2012 onwards*
*Report of the Work of the Lambeth and Southwark Public Health Team 2013–2015*
*Southwark and Lambeth Integrated Care Report 2016*
*Southwark Five-Year Forward View*: *Into Action 2017 Presentation Slide Pack*
*Southwark Council Priorities 2015*
*Social Prescribing & Expert Patient Programme Modelling NHS Southwark CCG (HES 2013/14*, *2014/15*, *2015/16 & 2016/17 data) Presentation Slide Pack*
*Southwark’s Primary and Community Care Strategy 2013/14–2017/18*.

The document selection and analysis were not designed to be exhaustive, rather to give a snapshot of considerations surrounding the study objectives.

These documents were analysed using a thematic framework pertaining to user/patient multimorbidity ‘journeys’, along with community resources and how these were broadly conceptualised within statutory and charity reporting locally. Thematic framework analysis, an approach used widely in large scale policy research and increasingly in multi-disciplinary health research [15], utilises a framework structure to summarise the data to directly answer research questions.

#### Community stories

Due to the time limitations of the scoping study not allowing for patient interviews to take place, we sought to capture information relating to patient journeys to multimorbidity by extracting patient stories relating to their chronic conditions from publicly available websites and leaflets. The sources consisted of online fora, case studies, reports, paper brochures and community websites in Lambeth and Southwark, with information posted since 2012 (where content was dated) included. Five Southwark ‘stories’ and four Lambeth stories were included in the data. Though demographic data were not always available alongside the stories, five stories were reported to come from women, four from men. Age was stated in four cases, ranging from 32 to 60 (mean average = 47). The information was analysed using a six-phase analytic framework [16] to elicit inductively the significant features associated with living and managing multiple long-term conditions and the role of community assets. The six phases involved: 1-Transcription, 2-Familiarisation with the data, 3-Coding, 4-Developing a working analytical framework, 5-Charting data into the framework matrix, and 6-Interpreting the data.

#### Stakeholder interviews

The interview recruitment process used purposive sampling, whereby professionals working in the community for both the statutory and charity sectors were approached. Of the eleven potential participants approached, five responded, with four recruited to the study. One participant worked across both Boroughs, one in Southwark, two in Lambeth. The participants included one local general practitioner and three community organisation staff members, two in middle management, one senior. All four participants identified as women.

The participants were sent a covering email with information about the study, including how their contributions would be used and information about their right to withdraw at any time. Before commencing the interview, participants were asked to complete a consent form. The interviews were semi-structured and the loose exploratory interview guide (which was adapted according to the themes elicited within each individual interview) aimed to explore the participants’ perceptions around multimorbidity and community assets. The questions are shown in Table 2.

**Table 2:** Semi-structured interview protocol.

○ *Tell me about your role and the type of work you do in the area of multimorbidity*.
○ *Tell me about your role and the type of work you do in the area of multimorbidity*.
○ *What do you think that people with three or more long-term conditions require in terms of community support*?
○ *What would be the barriers/opportunities in terms of seeking community resources for support with multimorbidity*?
○ *What general reflections would you have on multimorbidity*? *Patient experience/their ‘multimorbidity journey’/provision/how it is understood locally and beyond*?

The researcher took notes throughout the interviews to record data and preliminary reflections, which formed the basis for deciding the themes presented within the findings section.

The interview data were analysed iteratively for themes using the six-phase analytic framework, using codes to build overarching themes. The themes were refined through iterative discussion with the co-investigator and funder. There was insufficient time allowed for participant validation of themes.

### Ethics

The study, categorised as minimal risk, gained ethical approval from King’s College London Ethics Committee (reference MR/17/18-269). Much of the study used publicly available information as data. Therefore, confidentiality and anonymity were only necessary to address with the semi-structured interviews.

## Findings

Findings are presented in turn for each of the four numbered microstudies with illustrative quotes to convey the salient ideas and beliefs captured through the thematic analysis.

### 1. Rapid review of the literature

Overall, the findings pertain to a paucity of literature directly addressing the interaction between multimorbidity and community support, as well as to a lack of useful interdisciplinary frameworks for understanding the ‘multimorbidity journey’. It appears therefore that the role of community resources in the management of multimorbidity is under-researched and not widely understood. We aim to begin to bridge this gap through our analysis by identifying several key overarching themes from the literature surrounding patients’ self-perceptions, the role of policy and the need to re-conceptualise approaches to multimorbidity.

Key themes resulting from the review of the literature were as follows:

#### Theme 1: Role of attitudes and understandings in the management of MLTCs

Several studies found that how individuals conceptualise their multimorbidity influences perceptions of self and, in turn, the management of their condition(s). For instance, some patients may perceive their symptoms as stemming from one long-term condition in isolation, and others as the result of multimorbidity [17]. Similarly, another study argued that how patients understand and negotiate the complexities of living with chronic illness will underpin their help-seeking and self-management [18]. Additionally, the level of motivation and responsibility towards self-management that the patient holds, will likely affect their level of engagement with treatment [19].

A meta-synthesis of qualitative studies [20] discovered that multimorbidity is experienced as moments of complexity, rather than counts of illnesses. Further, patients saw engaging in behavioural strategies with a social or spiritual component as part of taking responsibility for leading a purposeful life beyond the immediate context of multimorbidity. Several studies reviewed called for a greater awareness of the social context and social support available to individuals with multimorbidity [21, 22]. One study highlighted the importance of patients’ sense of social connectedness across the life course and the interaction of this with their psychological resilience [23]. Further, some commentators argued that an awareness and reconceptualisation of how patients’ perceptions of their own multimorbidity are understood hold implications for care provision and intervention design in this population [24].

#### Theme 2: Moving beyond silos in the management of multimorbidity

This theme echoes the subsequent findings of the four remaining micro-studies. Overwhelmingly, the literature supported a vision whereby services are not fragmented by individual diseases, rather become integrated for a seamless multimorbidity pathway. There is a clear theme within the literature that deficient integration between care providers often leads to fragmentation and ineffectiveness, leaving many patients frustrated and unable to navigate their way across their disjointed care pathway towards better health outcomes [25].

Working in silos is not only viewed as unsatisfactory for the patient experience, but also an inefficient use of public funds [26]. Services should be truly integrated, with an awareness of social issues at their core, and goal rather than symptom-oriented [27]. Notably, practices with a non-disease specific multimorbidity focus showed more integration of services [28], suggesting a need for reconceptualising multimorbidity as a whole ‘greater than the sum of its parts’. For instance, it has been argued that NICE guidelines on the management of single long-term conditions may not be applicable to a MLTCs patient profile [29]. A change of structure and process is required for implementing change towards an approach more tailored to multimorbidity [30]. Overwhelmingly, the literature conveyed the role that policy can play in improved understanding of and provision for MLTCs [31], including a change in perspective on multimorbidity [32] and establishing greater continuity of care [33].

#### Theme 3: Community assets and long-term conditions

Generally, there was a paucity of papers which explored the use of community resources, though one can draw inferences from the themes above around the utility of community resources the management of multimorbidity. However, numerous papers explored the utility of integrated community support around a specific chronic condition, in particular addiction [34] and mental illness [35]. One paper [36] made a compelling case for the role of community resources in the management of multimorbidity, stating that “communities and voluntary organisations often contain the necessary energy and enthusiasm to make a difference. This can have dramatic effects on a whole community, improving a range of measures, including the care of long-term conditions”. In line with this, another paper [37] argued for community-embedded resources which address the needs of the patient within the context of their multimorbidity experience.

A framework was identified in the published literature designed to expand aspects of improving care for long-term conditions in the community [38], and recognised the importance of meeting local demand through community resource to address the primary concerns of the WHO. However, it is not unlikely that against the backdrop of austerity politics in England, meeting the demand for community services is increasingly challenging.

Lastly, straying from the original inclusion criteria for the rapid review, one quantitative paper [39] has been included from 2007 as it evaluates the effectiveness of an expert patient programme [40] through a randomised controlled trial and concluded “lay-led self-care support groups are effective in improving self-efficacy and energy levels among patients with long-term conditions.” This finding is relevant to the scoping study which identifies the expert patient programme as one of several resources available to patients in Lambeth and Southwark.

### 2. Documentary analysis

Overall, the findings indicate that there is recognition of a financial resource-poor climate, but with a parallel view of the value of community assets and engagement. Generally, the policy and guidance discourse does see a move away from a specific condition focus to approaches to multimorbidity more broadly.

#### Theme 1: Resource poor climate

Overwhelmingly, an austerity discourse featured within the documents analysed. The content of the documents often proposed service improvements in relation to doing the most possible against a backdrop of limited resource and pressure for cost-savings, along with identifying the financial burden of multimorbidity:

*“The recent welfare reforms*, *austerity measures and the economic downturn have affected disadvantaged communities the most*.*”* (Public Health Report Health in our Community–Southwark 2013–2014 [41])

*“Although people with LTCs only account for 30% of the population*, *it is estimated that they utilise around 70% of the healthcare budget*.*”* (Southwark Integrated Care for Long-term Conditions Presentation Slides [42])

*“Our vision was for local health and social care systems to work in partnership to improve the way care is provided in Southwark and Lambeth*, *so that local people’s needs are recognised and they can be supported to lead healthier and happier lives*. *And we had to do this while taking into account tough financial constraints*.*”* (Southwark and Lambeth Integrated Care Report 2016 [43])

Indeed, integrated care was viewed as a financially and economically sustainable solution:

*“Success for us will be a fully integrated system of care delivering better outcomes within a sustainable health and social care economy*.*”* (NHS Lambeth Clinical Commissioning Group “Healthier Together” Five-Year Strategy: 2014/15 to 2018/19 [44])

Though it appeared that care integration locally has not fully embedded:

*“We heard issues around the stress of chasing up medical and social care appointments (…) ‘(…) this week has been really difficult with people visiting all the time*, *carers*, *district nurses*, *physiotherapists*. *(…) It was so frustrating when a different carer visited and we had to explain everything all over again*.*’”* (Healthwatch Lambeth Annual Review 2016–2017 [45])

Further, community assets themselves, i.e. the Expert Patient Programme, were presented in local policy and guidance documents as generating return on investment and associated cost-savings:

*“(The aim is) to calculate the return to the NHS in London on investment in implementing (…) (the Expert Patient Programme) initiative over a five-year period*.*”* (Social Prescribing & Expert Patient Programme Modelling NHS Southwark CCG Presentation Slide Pack [46])

#### Theme 2: Recognising the role of community assets

Most reports recognised the role of the community and community assets either in terms of support and intervention around MLTCs or local public health more broadly, especially within a resource-poor climate and people affected by disadvantage.

Multimorbidity was conceptualised within the documents studied as being multi-factorial, along with stemming from a complex interplay of numerous causal factors with patients’ socio-economic status and disadvantage, and it was claimed that working at a community level could help tackle social factors:

*“Improving the wider determinants of health involves working with a wide variety of partners (…) engaging and involving communities by working with residents can help to achieve citizen-led activities that build on existing assets to address localised social determinants of health*.*”* (A Report of the Work of the Lambeth and Southwark Public Health Team 2013–2015 [47])

Further, a need for community engagement was recognised within the documents analysed, along with a sense of tangible actions to achieve it:

*“Community focus groups—we have held 5 focus groups over the year with different communities living in Southwark*:*—Bengali–Vietnamese—Gypsy and Traveller x3*.*”* (Healthwatch Southwark Annual Report 2015–2016 [48])

*“Connecting the community—we continue to gather experiences from those involved in the Lambeth Council and NHS Lambeth Clinical Commissioning Group’s Community Connectors programme*.*”* (Healthwatch Lambeth Annual Report 2016–2017 [49])

#### Theme 3: Solution-focused intentions

The reports were overwhelmingly solution-focused, identifying strategic actions to be taken to improve the health of the public locally, and centred around stated priorities. Multimorbidity featured highly in the discourse within the documentation analysis. Whilst there was focus on prevention for cost-savings and social factors related to multimorbidity, such as local health inequalities, the MLTC profile journeys were notably absent from the documents studied:

*“over the next five years our strategy aims to improve the health and wellbeing of people in Lambeth (…) reduce health inequalities across Lambeth and between Lambeth wards*.*”* (NHS Lambeth Clinical Commissioning Group “Healthier Together” Five Year Strategy: 2014/15 to 2018/19 [50])

*“Local health and care planning*: *Menu of preventative interventions Public Health England has worked with partners to identify preventative actions that can improve people’s health*, *support quality improvement and potentially save the NHS and the wider system money*.*”* (Commissioning for Value Long-term Conditions Pack–Southwark CCG 2016 [51])

*“We engaged in some focused patient insight work to help us to understand the nature of living with multiple long-term conditions*.*”* (Southwark Five Year Forward View: Into Action 2017 Presentation Slide Pack [52])

Next, the findings from the third micro-study are presented.

### 3. Community stories

The themes emerging from nine Southwark and Lambeth patient case studies, narratives and stories available in the public domain are presented, with illustrative excerpts. Five Southwark ‘stories’ and four Lambeth were examined. There were numerous parallels between both Southwark and Lambeth respondents, and no unique themes emerged in relation to just one Borough. As such, the themes are not organised as Borough-specific considerations.

#### Theme 1: Lack of joined-up approach

Chiming with the literature review theme of moving beyond silos in the management of multimorbidity, there were several examples within the sources analysed of interventions and support around MLTCs not being joined-up nor integrated, which led to poor patient service experience:

*“There wasn’t enough time to discuss the whole picture with the GP–even with a double appointment*. *I found it really difficult to get guidance and support and had to shout for any services I received”* (Story 1)

*“I needed help from someone who could look at me as a whole person*. *It’dd have really helped to have on-going support for my weight management to keep up my morale and give encouragement if I felt myself slipping back into old ways”* (Story 1)

*“There are so many consultants to see in different places*, *why can’t we just see them all on the same day*, *in the same place so we don’t waste time and energy*, *which often makes us feel even sicker*.*”* (Story 5)

The stories indicated how a more integrated approach to their care helps patients regain control over their appointments and medical care:

*“(He) also tries to have what he terms as a ‘medical free week’ by carefully scheduling appointments close together*, *where possible*, *so one week is free of appointments*.*”* (Story 4)

#### Theme 2: Loss of control vs. confidence

As well as the perceived lack of control resulting from a disjointed approach to the treatment of MLTCs, the stories conveyed a sense of loss of control in the sick role resulting from the perceived medical complexity surrounding multimorbidity:

*“(Name withheld)*, *aged 60*, *has had two heart attacks the first at 37 years of age*, *the second at aged 50*. *Sixteen years ago*, *(name withheld) was diagnosed with type 2 DM*. *He also has rheumatoid arthritis*, *diabetic neuropathy and vascular disease in both legs*. *When (name withheld) is with his physician*, *he rarely asks questions*, *and says that he just ‘sits and nods’ in agreement*. *(Name withheld) says that he doesn’t question the physician’s decisions; he trusts the medical professionals to make the right decisions for him*. *(Name withheld) doesn’t expect his doctors to go through all the technical medical terms with him as he wouldn’t understand them*. *He doesn’t understand the clinical language in his hospital letters*.*”* (Story 7)

There were several reflections suggesting that MLTCs led individuals to feel that they lack the ability to cope, causing a sense of personal failure:

*“Sometimes I felt like everything was getting on top of me and I felt ashamed that I couldn’t cope*.*”* (Story 3)

However, it seems that community resources have an important part to play in helping individuals with MLTCs regain a sense of control over their conditions and lives, as these testimonies linked to local community assets strongly suggest:

*“No matter what else is going on in my life*, *(the community resource) made me feel safe*, *secure*, *uplifted and equal*.*”* (Story 6)

*“I felt more self-worth and self-confidence*.*”* (Story 8)

*“(Name withheld) often has periods of self-reflection and feeling low*, *and questions why she has so much and why it’s always her who suffers*, *but does try to see a positive outcome and carry on with her life*.*”* (Story 9)

#### Theme 3: Mental wellbeing and hope through reducing isolation

The final theme pertained to the interplay between MLTCs and individuals’ mental health and wellbeing, and a perceived sense of hope offered by community organisations to their uses. It is suggested that mental and physical health are perceived by patients as being inextricably linked, affecting one another:

*“His physical ailments exacerbate his mental health problems*.*”* (Story 8)

*“(Name withheld) suffers from a serious of horrendous side effects*, *which include Jonson Steven’s Syndrome- a skin condition*, *Anaphylaxis*, *arrhythmia*, *ulcerative colitis and coeliac disease*. *(Name withheld) has also been diagnosed with intermittent depression*.*”* (Story 4)

Mental health problems were at times viewed as being the direct result of the MLTC diagnosis and a resulting inability to cope:

*“Tina used to work as a nurse in the army before being diagnosed with lupus in 1986*. *She had to leave her job and found it hard to come to terms with the diagnosis and civilian life*. *This led to inactivity and emotional eating to cope with her symptoms*.*”* (Story 3)

Moreover, there was a clear sense of community resources helping their members gain hope through reducing their perceived isolation and improving their mental and physical wellbeing and coping abilities:

*“The course meant the world to me*, *there’s nothing worse than losing hope*. *It’s given me a tremendous amount of hope that there’s a future and I can look forward to it*.*”* (Story 2)

*“Right from the first meeting I felt I wasn’t alone*. *I walked in alone*, *but afterwards*, *I felt more self-worth and self-confidence*. *It was great to meet like-minded people*, *with similar issues*. *It’s not just always the medical or physical condition*, *it’s about the feeling of being human and that you’re worth something*.*”* (Story 4)

Often this sense of hope led to tangible biopsychosocial change:

*“I’ve lost over two stone and I want to start working again*.*”* (Story 3)

### 4. Community stakeholder interviews

There were numerous parallels between both Southwark and Lambeth respondents, and no unique themes emerged in relation to just one Borough. As such, the themes are not organised as Borough-specific considerations.

#### Theme 1: Disjointed care and barriers to access

The stakeholders interviewed perceived the statutory care that those with MLTCs received to not be adequately joined-up, lacking a biopsychosocial perspective, and often affected detrimentally by the lack of funding:

*“Feedback from pilot (of care assessment) is currently disappointing–assessment hasn’t been explained well to people*, *doesn’t identify key goals which matter to them and how they’re coping with their conditions–been a tick-box exercise*, *without the wider life perspective being explored*. *The follow-up input hasn’t happened*, *not joined up at all*.*”* (Pp. 1)

*“Trying to maximise existing structures doesn’t allow to individualise who’s in on the discussion according to their needs*, *e*.*g*. *someone from housing*. *Seems to be the key problems for individuals–affects mobility*.*”* (Pp. 1)

Resources in the community were perceived as partly providing a solution to the disjointed statutory provision:

*“Care is very fragmented*. *Often the individual goes from appointment to appointment*, *they become almost a career patient*, *preoccupied with their conditions and organising their lives around disjointed services and appointments*. *Whereas resources in the community remove the medical aspect*, *so are less pathologising*, *more holistic*, *they help enhance the person rather than just focus down on symptoms or the individual diseases*. *They may also be good at serving a specific population*, *say culturally or services for women only*. *Sadly*, *we all know that there’re problems with funding*, *resources*, *but with chronic diseases on the increase*, *perhaps that more local*, *community way of looking at things is the way forward*.*”* (Pp. 2)

Closely echoing the findings of the other micro-studies, the participants reflected on how there is a perceived lack of a joined-up approach around service organisation for chronic diseases more broadly, even less so for multimorbidity. Further, the services which are viewed as community assets were often seen to not be well-marketed or accessible to statutory referrers. This was conceived as being difficult for practitioners and patients alike:

“Multimorbidity isn’t considered in the context of the whole-patient and services reflect this. Pathways aren’t even straightforward if you have just one long-term condition, let alone many.” (Pp. 4)

“I have to google what services are available to me as a referrer locally. They often aren’t well-promoted well from the community to us GPs. So, putting things out in a newsletter say isn’t sufficient and shows lack of understanding of the volume of work we encounter.” (Pp. 3)

### Theme 2: The importance of holism

Related to the theme of disjointed services and poor patient experience, holism (both as a philosophy and approach) was highlighted by all participants as having the potential to improve MLTC patients’ care experience:

*“If we carry on with our process-based culture*, *with numbers*, *we fail to help the people*.*”* (Pp. 3)

*“We did talk about needing longer appointment times for reviews so that we can pay attention to social and psychological factors*, *but we haven’t done anything about it*. *We must remember that not every consultation is the same; some people are well-managed*, *others are on the other end of the spectrum*. *Often*, *lack of time and resources doesn’t allow us to approach our patients in such a tailored way*.*”* (Pp. 3)

*“Social issues (loneliness*, *isolation*, *family*, *relationships*, *neighbours) are so important in people’s lives*, *especially if living with several health issues*, *but this wasn’t covered in the assessment–how someone’s life is*, *how they are coping*, *emergency planning were all missing*.*”* (Pp. 1)

*“Allowing time to hear people’s stories is so important*, *learning about their context*, *rather than jumping to solve problems in a biomedical model*.*”* (Pp. 1)

Further, there was a sense of how the holistic approach should encapsulate an insight into the patient ‘journey’ towards MLTCs:

*“What is it that gets people there*? *Well it’s poor health behaviours*, *genetics*, *stressful life events*, *we probably shouldn’t underestimate how much life stress can play into all of this*.*”* (Pp. 2)

*“We’re here to provide a voice to people of different ages and backgrounds*, *at different life stages*, *what they’ll require from a service to help them deal with their diseases is so dependent on that and I’m not sure we can ever truly represent all the unique needs*, *but we can try by being sensitive to their life stories*, *what they are saying about how their chronic diseases affect them*.*”* (Pp. 4)

### Theme 3: Community opportunities

Community assets were discussed by the participants as providing rich opportunities to deliver a more holistic approach in terms of addressing socio-psychological factors, outside of, or even beyond the biomedical model, driven by bottom-up needs and motivations:

*“There’s always something tailored about approaches which are designed by the people for the people*.*”* (Pp. 4)

*“Getting support in the community can be invaluable*, *as it’s just something you do as part of your day*, *to connect with others; it isn’t stigmatising*, *it’s not about pills or diagnosis often*, *just getting together with others for a coffee and a chat*.*”* (Pp. 2)

Echoing the theme of ‘mental wellbeing’ stemming from the community stories, it was felt by the interviewees that community assets have an important part to play in providing support for individuals diagnosed with mental health:

*“We especially need community assets for people with chronic diseases and mental health problems*. *We all know that those with severe mental illness tend to die earlier due to chronic conditions and their physical health gets ignored*.*”* (Pp. 3)

## Discussion

There are several parallels to be drawn across the four micro-studies in terms of how multimorbidity is understood locally and beyond. The micro-studies covered twelve themes as detailed in the result section and of these multiple themes, theme 2 ‘The need for holism’ (of micro-study four) appears to be an overarching theme as it encapsulates nearly all other themes. In fact, the findings indicate that multimorbidity is multifactorial and complex and a single disease focus is unhelpful, as is the perceived resulting lack of joined-up service approach to multimorbidity. The theme calls for a joined-up and holistic approach to multimorbidity and patient care which surfaced as an issue of heightened importance. This theme ran across the four micro-studies, highlighting important implications for not only practice, but also for service (re)organisation [53]. Gouin & Kiecolt-Glaser [54] for instance found that healing is both psychological and physical, reinforcing a well-documented perspective in medical practice and the sociology of health and wellbeing (see also Nunes [55]; Parsons [56])

It is reassuring that the goal of integration features highly within the local policy and guidance literature across the two Boroughs. However, there is a sense within the community stories and stakeholder interviews that integrated care has not been embedded as successfully as expected, with patients voicing their dissatisfaction with the lack of coherent care around their multiple conditions. This closely echoes the findings of the Southwark and Lambeth Integrated Care (SLIC) report, which highlights that the delivery of care could not be integrated unless the systems underpinning it were also integrated as a key milestone in their programme [57, 58].

The lessons from Southwark and Lambeth may be that a reconceptualisation of multimorbidity is commanded before meaningful integration. Several findings within this scoping study suggest the need to consider the journey towards multimorbidity, in terms of recognising life course factors in order to design appropriate interventions. A number of studies reviewed as part of this work called for greater awareness of the social context and social support available to individuals with multimorbidity. The work of Meleis [59] and others has relevance; patients were identified as being more at risk of poor health due to the vulnerability linked to a transition point in their lives. This paper suggested that further work around the ‘multiple dimensionality’ of transition points and social experiences is needed to guide practice.

Some commentators propose that awareness and reconceptualisation of how patients’ perceptions of their own multimorbidity are understood hold implications for care provision and intervention design. McSharry and colleagues [60] argued that more understanding needs to be gained beyond a single disease focus, and that the composition and interaction of multimorbid presentations is of greater importance when working with patients. Indeed, their paper found that patients’ representations of their own illness were varied, with some seeing the management of their individual conditions as in complement, others as in conflict. The authors saw utilising patients’ individual understandings as key in more effective management of their condition. Coventry and colleagues [61] suggested that an understanding of the lived experience of multimorbidity is crucial to designing and delivering interventions. The authors promoted the role of appropriate social and psychological support in facilitating self-determination, whilst arguing for a reconceptualisation of multimorbidity which recognises the role of self-agency in patients leading a fulfilling life.

Providing more support towards rethinking how multimorbidity is understood, Haggerty [62] argued for its reconceptualisation, which would form the basis for better organisation and greater continuity patient care. The public health concept of ‘syndemics’ well used in medical anthropology. Syndemics frameworks examine how diseases interact with social, environmental and economic factors that mitigate disease. As a reflection of this call for reconceptualisation, syndemics can help reduce health inequalities [63]. There is evidence that minorities and socio-economically disadvantaged cluster often have multiple coexisting underlying conditions which place them in the high-risk categories with regards to mortality. As it is well-known that health inequalities are linked to the unequal encounter with socio-economic determinants, the syndemics approach would command action on such determinants in the form of public health education, community engagement and early intervention [64].

The findings of this scoping study underline the significance of considerations of psychosocial factors [65–67] feeding into support needs of individuals with MLTCs, including their motivations to engage with disease management, their self-identities and their experiences beyond the biomedical model. Each of the themes provides significant support for this analysis. For instance, the community stories conveyed a feeling of control lost through the experience of multimorbidity. This may be linked to a shaky perception of ‘psychological ownership’ [68] influenced by individuals’ lack of perceived capacity to control the course of their illness, perpetuated by a disempowering experience of the health system (e.g. multiple appointments dealing with one aspect of their condition at a time at several sites). The reported journey of a move away from control loss towards confidence reported in this study can be viewed in the context of ‘integration’ [69]. Integration represents the process undertaken by an individual to integrate their chronic illness into their daily life, including tasks of self-management and treatment, without their identities becoming subsumed by a ‘sick role’, enabling them to continue to participate in a self-defined meaningful life. The individual and contextual factors which facilitate integration require further study to help inform future interventions. Person-centredness and holism within the biopsychosocial framework are key in treatment approaches and designs. This is nothing new—numerous papers [70, 71] argue for a holistic, person-centred care; however, the whole-person approach is more critically significant for multimorbidity management.

Social factors in particular featured strongly across the four studies as playing a part in multimorbidity, be it as a causal factor, one influencing patient journeys, or the management of their MLTCs. This perspective confirms Parsons’ [72] assumption that the sick role is played in social and psychological contexts. It is apparent from the study that community assets have an important and recognised role to play in supporting those with multimorbidity holistically in a non-stigmatising way. They are perceived to go a long way towards meeting the social needs of people with MLTCs and a way of ‘filling the gaps’ in statutory provision against an enduring backdrop of austerity politics. Community assets also lend support beyond the biomedical paradigm. This echoes calls for a move away from a disease-specific approaches to multimorbidity [73, 74].

Finally, there are several considerations stemming from the overarching findings which may hold implications as to the types of approaches taken to multimorbidity in local regions. For instance, given the recognised utility of community assets in the support for people with MLTCs, in particular social support, it is important to ask the question as to how one goes about designing/implementing/strengthening community assets. Here, it is apt to mention Stroul’s framework of the community support system (CSS) [75], initially used to map the components of a community support system for someone living with mental illness, to identify the crucial components required to build a beneficial community asset. CSS values include family and community involvement, and flexible services, with cross-system collaboration.

According to Fleury and colleagues [76], a community support system ought to be integrative and culturally relevant. Lipsey’s program theory [77] is one operationalisable framework in the literature which could form the basis of designing a community asset programme by considering several key factors elicited through this scoping study; namely—the problem of demand for integrated holistic multimorbidity provision; the mediating processes such as individual attitudes and psychosocial factors; the expected outcomes in terms of perceived wellbeing; and implementation considerations including issues of limited financial resource and marketing of provision.

### Study limitations & further work

Rapid ethnography, the method employed in this study, has several limitations. The key limitation in terms of this study is related to its time constraints. There was limited time to engage community stakeholders and patients with this study, and therefore, their receptiveness to it is likely to have been minimal, which may impact on the validity of some of this study’s findings. It is unlikely that a data saturation point has been reached, where no new findings emerge, though a scoping study is designed to act as an introductory exercise into broader future work. Nonetheless, this limitation was mitigated by the rapid ethnography approach drawing on a range of perspectives about MLTCs and community assets by facilitating a timely integration of multiple data sources, eliciting a series of rich themes holding several implications for practice.

Future work speaking to patients and members of the public directly would help gain a sense of the types of community assets that are either engaged with by people with MLTCs or that there is a demand for. Further, it would be of interest to explore with community stakeholders what the barriers to establishing and sustaining local assets for MLTCs may be. This insight may potentially help to inform the funding priorities for future programmes.

In terms of the documentary analysis, the selection of local policy and guidance documents was opportunistic and limited to online search engine results. The approach to organising the documentation by type and time period was not systematic and numerous key reports may have been missed. It is also difficult to judge the extent to which these reports have an impact locally. Future work would benefit from a more systematic approach to sourcing and analysing policy documentation, potentially along with publicly available meeting minutes to gauge the extent of their influence. Nonetheless, the limitation of the documentary analysis was mitigated as the authors brought the content of several documents together into overarching themes; in addition, with the documents accounted for in the study, no new findings pertaining to MLTCs and community assets emerged, suggesting that a saturation point had been reached.

In terms of limitations of the study, we acknowledge that though the focus was on community stories and assets, some patient/user interviews would enhance the study by putting some of the findings in perspectives, providing basic lines of enquiry for further investigation [78]. The reason for this was the time limited nature of the study, not allowing sufficient time for ethics application and approval and participant recruitment. However, the community stories presented here go some way towards conveying the patient ‘voice’, though it is worth noting that several stories included in the study were written in the third person, which suggests that they may have been a subjective interpretation of a patient’s experience by another. Further, one of the original objectives of this study was to reconstruct the patient ‘journey’ towards multimorbidity. The data analysis largely did not identify themes pertaining to the patient journey and it is likely that it is only through speaking directly to patients that conceptualisations and constructs of the trajectory towards MLTCs can be truly captured.

Further qualitative work could shed light on the types of health beliefs and behaviours which individuals perceive in retrospect to have been a catalyst for their subsequent chronic diseases, and the types of behaviours which also contributed to their wellbeing, along with the role community assets may have in the avoidance or management of treatable risk factors.

## Conclusions

By utilising a rapid ethnographic approach and bringing together multiple data sources from a series of micro-studies, the study has provided an insight into some of the key themes surrounding MLTCs in Lambeth and Southwark and beyond. These two inner London boroughs have significant minority populations and pockets urban poverty; this makes the results of the study relevant to other urban settings which may present similar characteristics of urban multimorbidity and community assets. To revisit the original aims of this scoping study, the overall conclusion pertaining to ‘reconstructing’ the journey towards multimorbidity in Lambeth and Southwark is that there is indeed a sense of there being a journey, punctuated by life events which may play into patients’ experiences of MLTCs, and further work to explore the meanings surrounding those life events, along with appropriate tailored interventions, is recommended. In terms of perceptions of community assets, overwhelmingly community resources are welcomed and seen as non-stigmatising and holistic in their non disease-specific approach and as having a key part to play in support for people with MLTCs. However, multimorbidity resource remains patchy and working within a narrow demand area. In terms of service provision, there remains a call for services which are not disjointed and treat the whole person, rather than each of their long-term conditions in isolation.

Multimorbidity is a complex phenomenon is terms of how it is experienced and understood by patients, clinicians, community stakeholders and policy makers. This study has contributed in scoping some of the attitudes and perceptions towards MLTCs in communities to help to begin to reconceptualise the way multimorbidity is understood and managed locally in urban settings and beyond.
